# The Efficacy of Various Pharmacological Agents on Long-Term Outcomes in Patients With Heart Failure With Preserved Ejection Fraction: A Meta-Analysis of Randomized Control Trials

**DOI:** 10.7759/cureus.28145

**Published:** 2022-08-18

**Authors:** Sana Faisal, Zubair Ahmad Ganaie, Saima Batool, Dr Hatim I Lokhandwala, Joseph Hankins, Sandipkumar S Chaudhari, Rimsha R Vohra, Shamsha Hirani

**Affiliations:** 1 Family Medicine, Dow University of Health Sciences, Karachi, PAK; 2 Internal Medicine, Holy Family Red Crescent Medical College, Srinagar, IND; 3 Internal Medicine, Hameed Latif Hospital, Lahore, PAK; 4 Internal Medicine, Jinnah Postgraduate Medical Centre, Karachi, PAK; 5 Faculty of Medicine, Universidad Latina de Costa Rica, San Pedro, CRI; 6 General Practice, General Hospital, Becharaji, IND; 7 Internal Medicine, Dow Medical College, Karachi, PAK; 8 Cardiology, Baqai Hospital, Karachi, PAK

**Keywords:** cardiovascular mortalit, all-cause mortality, cardiovascular outcomes, meta-analysis, heart failure with preserved ejection fraction (hfpef)

## Abstract

The beneficial impacts of various drugs on long-term outcomes in patients with heart failure with preserved ejection fraction (HFpEF) have been a matter of controversy. The aim of this meta-analysis was to systematically review randomized control trials (RCTs) involving patients with heart failure with preserved left ventricular ejection fraction (LVEF) and identify the effects of various treatment options [angiotensin-converting enzyme (ACE) inhibitors, beta-blockers, angiotensin receptor blockers, and aldosterone receptor blockers] on all-cause mortality, cardiovascular mortality, and hospitalization due to cardiovascular reasons. The current meta-analysis has been conducted as per the Preferred Reporting Items of Systematic Reviews and Meta-Analyses (PRISMA) guidelines. A comprehensive literature search was performed without any restrictions on language by using the electronic databases Cochrane Library, EMBASE, and PubMed up to July 20, 2022. The outcomes assessed in this meta-analysis included all-cause mortality, cardiovascular mortality, and hospitalization due to cardiovascular reasons. Overall, 10 articles were included in the current meta-analysis with a pooled sample size of 13,336 patients with HFpEF. In comparison to the placebo, among all four pharmacological agents, beta-blockers were the only agent that decreased the risk of all-cause mortality and cardiovascular outcomes. On the other hand, a significant reduction in hospitalization due to cardiac-related reasons was reported in patients on ACE inhibitors as compared to placebo. No other pharmacological agent had an impact on hospitalization due to cardiac-related reasons. The current meta-analysis indicates the possible benefits of beta-blockers in HFpEF in terms of reducing cardiovascular death and all-cause mortality.

## Introduction and background

Heart failure is a leading cause of hospitalization and it is associated with an increased burden on healthcare overall [1]. Heart failure with preserved left ventricular (LV) ejection fraction (HFpEF) is defined as the presence of heart failure without any evidence of decreased LV ejection fraction [2]. The prevalence of HFpEF is on the rise and it is associated with increased hospitalization rates [3]. Despite several advances in its management, numerous studies on patients with chronic heart failure have shown that this syndrome carries high mortality and morbidity rates [4].

Although evidence from trials demonstrating improvements in mortality has been inconsistent and largely neutral, several trials have suggested that drug therapy may improve exercise tolerance and quality of life in these patients [5]. Since patients with heart failure with preserved left ventricular ejection fraction (LVEF) are more likely to be older and tend to have more comorbidities compared to their counterparts [6], the effects of drug treatment might best be assessed by their impact on hospitalization and symptoms as well.

A randomized control trial (RCT) was conducted by Aronow et al. in 1997 to analyze the impact of propranolol (beta-blocker) on the risk of nonfatal myocardial infarction and mortality in patients with HFpEF. The study concluded that the risk of nonfatal myocardial infarction and mortality was lower in patients receiving propranolol as compared to patients in the placebo group [7]. On the other hand, the PARAGON trial found that the use of angiotensin receptor-neprilysin inhibitors in patients with HFpEF did not lead to any significant reduction in cardiovascular mortality and hospitalization as compared to the placebo group [8]. Due to inconsistent or lack of adequate evidence regarding the benefits of drugs in patients with HFpEF, it is important to review all the available treatment options to compare their efficacy in order to prevent severe outcomes in patients with HFpEF.

There is a need to comprehend how the effectiveness of these individual treatments and various combinations compare in terms of all-cause mortality and cardiovascular mortality. Given that the majority of trials on HFpEF have compared newer agents to placebo, alternative background treatments as recommendations have evolved. In this current meta-analysis, we aimed to systematically review RCTs involving patients with heart failure with preserved LVEF and identify the effects of various treatment options [angiotensin-converting enzyme (ACE) inhibitors, beta-blockers, angiotensin receptor blockers, and aldosterone receptor blockers] on all-cause mortality, cardiovascular mortality, and hospitalization due to cardiovascular reasons.

## Review

Methodology

The current meta-analysis has been conducted as per the Preferred Reporting Items of Systematic Reviews and Meta-Analyses (PRISMA) guidelines.

Data Sources and Searches

A comprehensive literature search was performed without any restrictions on language by using the electronic databases Cochrane Library, EMBASE, and PubMed up to July 20, 2022. In addition, bibliographies of relevant meta-analyses and studies were also searched. The search strategy included a combination of the following keywords: “heart failure with preserved ejection fraction”, “beta-blockers”, “ACE inhibitors”, “angiotensin receptor blocker”, “aldosterone receptor blockers”, “randomized control trial”, “cardiovascular outcomes”, and “mortality”. The EndNote software version X9 was used throughout the search and the screening process. Firstly, all searched articles were reviewed and duplicates were removed, which was followed by a title and abstract screening of the remaining articles. Finally, full texts of all the eligible studies were obtained and screened for inclusion and exclusion criteria. No restrictions were placed on the year and language of publication.

Study Selection

The literature search was carried out by two authors independently. In case of any disagreement, a consensus was reached through discussion. A study was eligible if the following criteria were fulfilled: (a) RCTs evaluating the effect of ACE inhibitors, beta-blockers, angiotensin receptor blockers, and aldosterone receptor blockers; (b) studies assessing at least one of the following three outcomes - all-cause mortality, cardiovascular mortality, and hospitalization due to cardiovascular reasons. Trials with a follow-up period of less than one month were excluded. Studies conducted on healthy volunteers or individuals with diseases other than HFpEF were also excluded. Lastly, studies that compared different doses of drugs were excluded from the current meta-analysis.

Data Extraction

An electronic data extraction form was created on Microsoft Excel and used for documenting basic data such as the first author's name, year of publication, intervention, the sample size in each group, follow-up duration, and outcomes. Two authors independently extracted the data and the data of both authors were compared. In case of any disagreement, a consensus was reached via discussion. If required, a third author was also involved in it.

Quality Assessment

The Cochrane Risk of Bias tool was utilized for assessing the methodological validity of all included studies. Chosen articles were scored while extracting data and RevMan version 5.4.0 was utilized to generate a quality assessment graph.

Data Analysis

Data analysis was performed using RevMan version 5.4.0 and STATA version 16.0. The Mantel-Haenszel method fixed or random effect model was used for estimating pooled risk ratio along with the 95% confidence interval (CI) for each of the three outcomes. A p-value <0.05 was considered statistically significant. RCTs with no outcomes events reported in the study groups were excluded from the analysis of that outcome event as they did not contribute to the risk ratio. Forest plots were used to present the risk ratio graphically along with 95% CI. I2 statistics were used to determine the heterogeneity between study results.

Results

**Figure 1:**
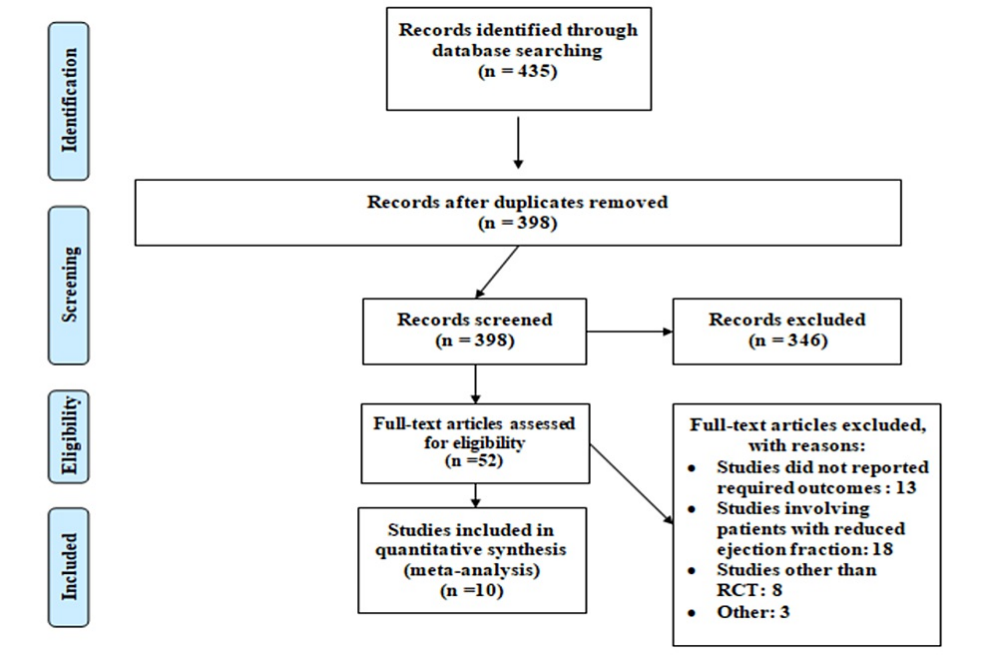
PRISMA flowchart of selection of studies PRISMA: Preferred Reporting Items for Systematic Reviews and Meta-Analysis

The PRISMA chart of the selection of studies is shown in Figure 1. Overall, 435 studies were identified through a systematic search using online databases. After removing duplicates, abstract and title screening of 398 studies were done. Only 52 studies were eligible for full-text review. Finally, 10 articles were included in the current meta-analysis. Table 1 shows the characteristics of all included studies, involving 13,336 patients with HFpEF. Four articles assessed the efficacy of beta-blockers [7,9-11], two assessed ACE inhibitors [12-13], two assessed angiotensin receptor blockers [14-15], and two assessed aldosterone receptor blockers [16-17]. The mean follow-up period in all included studies ranged from six months to 49.5 months.

**Table 1:** Characteristics of included studies ACE: angiotensin-converting enzyme

Study	Year	Interventions	Drug	Sample size	Follow-up
Aronow et al. [7]	1997	Beta-blockers	Propranolol	79	30 months
		Placebo		79	
Bergström et al. [9]	2004	Beta-blockers	Carvedilol	47	6 months
		Placebo		50	
van Veldhuisen et al. [10]	2009	Beta-blockers	Nebivolol	320	24 months
		Placebo		323	
Yamamoto et al. [11]	2014	Beta-blockers	Carvedilol	120	36 months
		Placebo		125	
Cleland et al. [12]	2006	ACE Inhibitors	Perindopril	424	12 months
		ACE Inhibitors		426	
Davis et al. [13]	2008	ACE	Lisinopril	98	20 months
		Placebo		227	
Yusuf et al. [14]	2003	Angiotensin receptor blocker	Candesartan	1,514	36 months
		Placebo		1,509	
Massie et al. [15]	2008	Angiotensin receptor blocker	Irbesartan	2,067	49.5 months
		Placebo		2,061	
Edelmann et al. [16]	2013	Aldosterone receptor blocker	Spironolactone	213	12 months
		Placebo		209	
Pitt et al. [17]	2014	Aldosterone receptor blocker	Spironolactone	1,722	40 months
		Placebo		1,723	

Risk of Bias Assessment and Publication Bias

**Figure 2:**
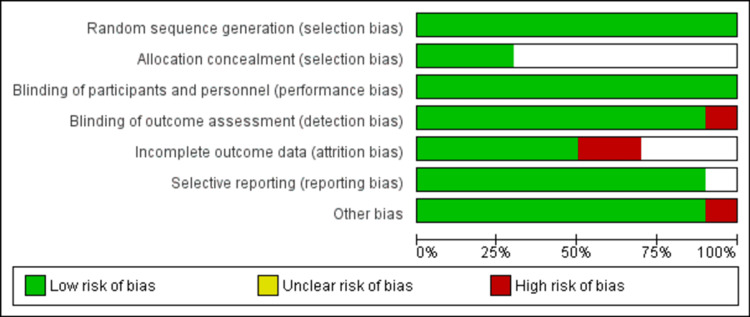
Risk of bias assessment

Figure 2 shows the risk of bias graph. Among 10 included studies, six had a low risk of bias and two had a moderate risk of bias. The remaining two studies had a high risk of bias.

Efficacy Outcomes

**Figure 3:**
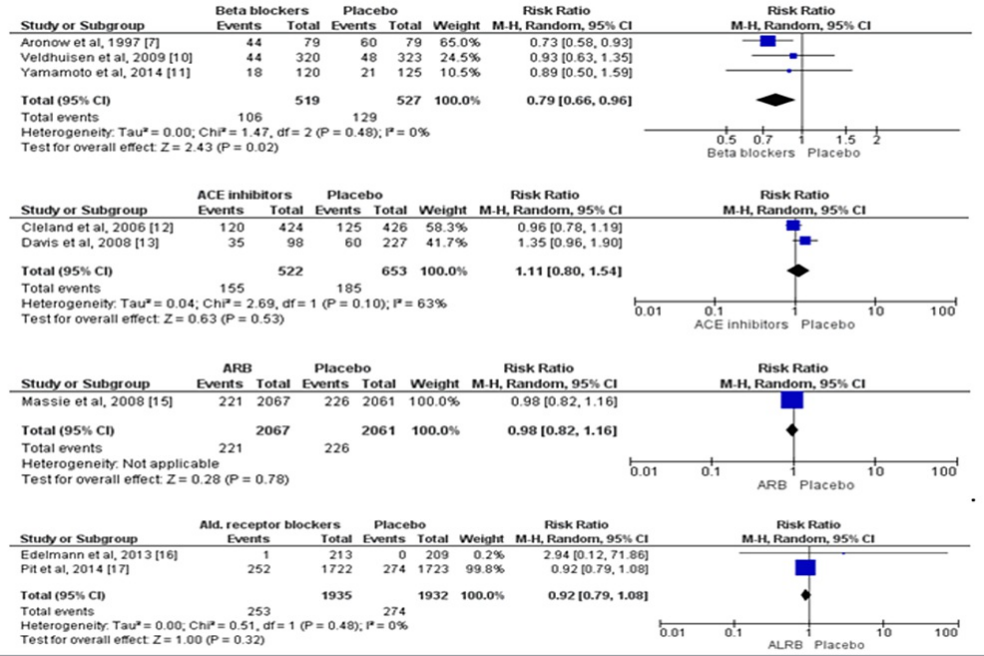
Pairwise comparison of each of the treatment groups with placebo for all-cause mortality* *[7,10-13,15-17] The figure depicts individual and pooled estimates of risk ratio along with their 95% CI for all-cause mortality for various therapies including beta-blockers, ACE inhibitors, angiotensin receptor blockers, and aldosterone receptor blockers ALDB: aldosterone receptor blockers; ARB: angiotensin receptor blockers; ACE: Angiotensin-converting enzyme

Eight trials involving 10,313 participants provided the outcome data for all-cause mortality [7,10-13,15-17]. The detailed pairwise comparison of each of the treatment groups with placebo for all-cause mortality is shown in Figure 3. In comparison to the placebo, among all four pharmacological agents, beta-blockers were the only agent that significantly reduced the risk of all-cause mortality. The risk of all-cause mortality was 21% lower in patients who received beta-blockers as compared to patients who received placebo (RR: 0.79, 95% CI: 0.66-0.96).

**Figure 4:**
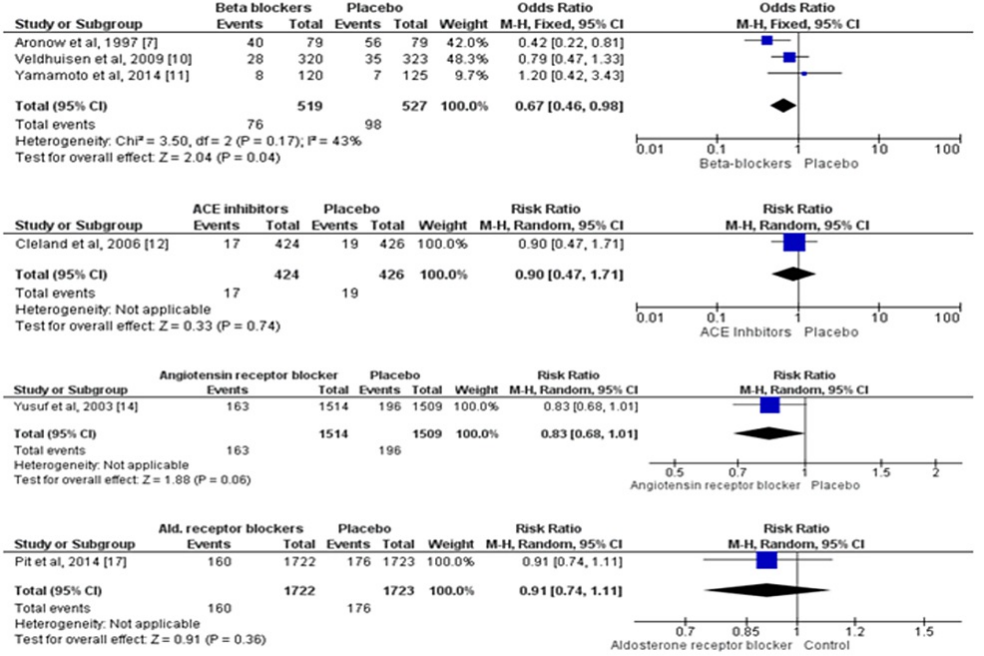
Pairwise comparison of each of the treatment groups with placebo for cardiovascular mortality* *[7,10-12,14,17] The figure depicts individual and pooled estimates of risk ratio along with their 95% CI for cardiovascular mortality for various therapies including beta-blockers, ACE inhibitors, angiotensin receptor blockers, and aldosterone receptor blockers ALDB: aldosterone receptor blockers; ARB: angiotensin receptor blockers; ACE: angiotensin-converting enzyme

Five RCTs assessed the impact of pharmacological agents on cardiovascular mortality among patients with HFpEF [7,10-12,14,17]. A total of 8,206 patients were enrolled in these five trials. Cardiovascular mortality was 33% lower in the beta-blockers group as compared to patients in the placebo group (RR: 0.67, 95% CI: 0.46-0.98). On the other hand, no significant impact of ACE inhibitors, angiotensin receptor blockers, and aldosterone receptor blockers was seen on cardiovascular mortality compared with controls, as shown in Figure 4.

**Figure 5:**
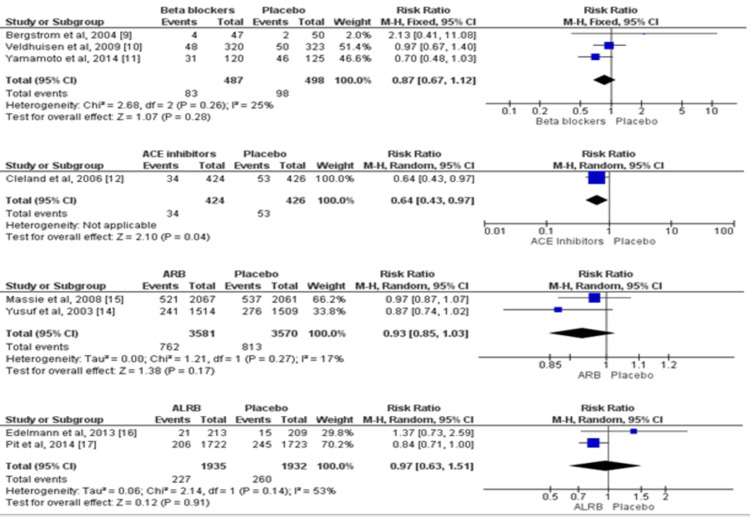
Pairwise comparison of each of the treatment groups with placebo for hospitalization due to cardiac issues* *[9-12,14-17] The figure depicts individual and pooled estimates of risk ratio along with their 95% CI for hospitalization due to cardiac issues for various therapies including beta-blockers, ACE inhibitors, angiotensin receptor blockers, and aldosterone receptor blockers ALDB: aldosterone receptor blockers; ARB: angiotensin receptor blockers; ACE: Angiotensin-converting enzyme

Eight RCTs assessed the impact of pharmacological agents on hospitalization due to cardiac issues among patients with HFpEF. No effect of beta-blockers, angiotensin receptor blockers, and aldosterone receptor blockers was seen on hospitalization due to cardiac-related reasons, as shown in Figure 5. However, lower hospitalization due to cardiac reasons was observed in patients receiving ACE inhibitors (RR: 0.64, 95% CI: 0.43-0.97).

Discussion

Based on our comprehensive meta-analysis of the available RCTs, beta-blockers are associated with a significant reduction in cardiovascular mortality and all-cause mortality in patients with HFpEF. However, ACE inhibitors, angiotensin receptor blockers, and aldosterone receptor blockers did not show any significant impact on all-cause mortality and cardiovascular mortality in the current meta-analysis. The impact of beta-blockers on mortality showed favorable outcomes in HFpEF patients. Similar results have been shown in a meta-analysis conducted by Liu et al. including both observational studies and previous meta-analyses [18].

The pathophysiology of HFpEF is heterogeneous, with several individual mechanisms coexisting within the same individual to cause symptomatic heart failure. Possible pathophysiological mechanisms include (a) thickness of the LV wall and/or expansion of the left atrium, both signs of increased ventricular filling pressure, (b) pulmonary vascular dysfunction or disease and failure of the right ventricle, and (c) plasma volume expansion [19-20]. The beta-blocker administration has been reported to have beneficial impacts on LV hypertrophy and fibrosis in an animal model of hypertensive diastolic heart failure [21]. The beneficial impacts of beta-blocker on LV hypertrophy and fibrosis may positively impact HFpEF because fibrosis and hypertrophy of left ventricles can contribute to the pathogenesis of HFpEF [22]. Finally, the protective effects of beta-blockers on oxidative stress and inflammation may increase the longevity of HFpEF [23]. One experimental study on hypertensive diastolic heart failure in a rat model found that the treatment with beta-blockers (bisoprolol) reduced inflammatory alterations and oxidative stress, which increased survival rates [24].

The European Society of Cardiology guidelines state that it is important to decrease the burden of readmission for patients with HFpEF [25]. Because patients with HFpEF are more likely to be older than those with heart failure with reduced ejection fraction (HFrEF), they have a higher burden of hospitalization for cardiac reasons, which is associated with a lower quality of life and a higher mortality rate [26]. In one of the studies included in the current meta-analysis, ACE inhibitor (perindopril) was significantly associated with decreased risk of hospitalization compared to placebo.

Choosing efficient medical therapies for HFpEF patients remains a major challenge for physicians. In this meta-analysis, we found that ACE inhibitors, angiotensin receptor blockers, and aldosterone receptor blockers failed to decrease the risk of mortality in these patients. The study conducted by Aronow et al. showed a positive result in decreasing mortality in patients with LVEF ≥40% [7]. Moreover, the renin-angiotensin-aldosterone system (RAAS) is usually the foundation of the evidence-based treatments to reduce mortality and morbidity in HFrEF patients [27], but there is no concrete evidence to suggest that they can improve the prognosis of these patients [28]. Hence, medications focused on this pathway will not improve mortality as RAAS activity is lower in HFpEF. Instead, the pathophysiology of HFpEF is primarily driven by tissue congestion brought on by elevated heart-filling pressures [29].

The current meta-analysis has certain limitations. Firstly, no data were available comparing different therapies with each other, and hence we were not able to compare the therapies with each other. Secondly, HFpEF is defined as heart failure in patients with LVEF of less than 50%. However, in some trials on HFpEF, patients with LVEF of 40-49% were usually included. Thirdly, risk ratios were used to determine the association as only a small number of studies reported hazard ratios (HR), which may cause bias associated with comparing outcomes in RCTs of various lengths. In the future, more RCTs need to be conducted with stringent criteria (ejection fraction of ≥50%) that compare different therapies with each other in terms of different outcomes including functional ability and quality of life along with cardiovascular outcomes in patients with HFpEF.

## Conclusions

As per our meta-analysis of RCTs involving patients with HFpEF, beta-blockers were found to decrease cardiovascular mortality and all-cause mortality. However, no significant effect of angiotensin receptor blockers, aldosterone receptor blockers, and ACE inhibitors on cardiovascular mortality and all-cause mortality was reported. Further studies need to be conducted to compare the efficacy of different pharmacological therapies in reducing cardiovascular events in patients with HFpEF.

## References

[1] Ponikowski P, Voors AA, Anker SD (2016). 2016 ESC Guidelines for the diagnosis and treatment of acute and chronic heart failure: the task force for the diagnosis and treatment of acute and chronic heart failure of the European Society of Cardiology (ESC) developed with the special contribution of the Heart Failure Association (HFA) of the ESC. Eur Heart J.

[2] Mozaffarian D, Benjamin EJ, Go AS (2015). Heart disease and stroke statistics--2015 update: a report from the American Heart Association. Circulation.

[3] Jaarsma T, Van der Wal MHL, Lesman-Leegte I (2008). Effect of moderate or intensive disease management program on outcome in patients with heart failure. The Coordinating Study Evaluating Outcomes of Advising and Counseling in Heart failure (COACH). Arch Intern Med.

[4] Owan TE, Hodge DO, Herges RM, Jacobsen SJ, Roger VL, Redfield MM (2006). Trends in prevalence and outcome of heart failure with preserved ejection fraction. N Engl J Med.

[5] Holland DJ, Kumbhani DJ, Ahmed SH, Marwick TH (2011). Effects of treatment on exercise tolerance, cardiac function, and mortality in heart failure with preserved ejection fraction. A meta-analysis. J Am Coll Cardiol.

[6] Mentz RJ, Kelly JP, von Lueder TG (2014). Noncardiac comorbidities in heart failure with reduced versus preserved ejection fraction. J Am Coll Cardiol.

[7] Aronow WS, Ahn C, Kronzon I (1997). Effect of propranolol versus no propranolol on total mortality plus nonfatal myocardial infarction in older patients with prior myocardial infarction, congestive heart failure, and left ventricular ejection fraction > or = 40% treated with diuretics plus angiotensin-converting enzyme inhibitors. Am J Cardiol.

[8] Solomon SD, McMurray JJ, Anand IS (2019). Angiotensin-neprilysin inhibition in heart failure with preserved ejection fraction. N Engl J Med.

[9] Bergström A, Andersson B, Edner M, Nylander E, Persson H, Dahlström U (2004). Effect of carvedilol on diastolic function in patients with diastolic heart failure and preserved systolic function. Results of the Swedish Doppler-echocardiographic study (SWEDIC). Eur J Heart Fail.

[10] van Veldhuisen DJ, Cohen-Solal A, Böhm M (2009). Beta-blockade with nebivolol in elderly heart failure patients with impaired and preserved left ventricular ejection fraction: data from SENIORS (Study of Effects of Nebivolol Intervention on Outcomes and Rehospitalization in Seniors With Heart Failure). J Am Coll Cardiol.

[11] Yamamoto K, Origasa H, Hori M (2013). Effects of carvedilol on heart failure with preserved ejection fraction: the Japanese Diastolic Heart Failure Study (J-DHF). Eur J Heart Fail.

[12] Cleland JG, Tendera M, Adamus J, Freemantle N, Polonski L, Taylor J (2006). The perindopril in elderly people with chronic heart failure (PEP-CHF) study. Eur Heart J.

[13] Davis BR, Kostis JB, Simpson LM (2008). Heart failure with preserved and reduced left ventricular ejection fraction in the antihypertensive and lipid-lowering treatment to prevent heart attack trial. Circulation.

[14] Yusuf S, Pfeffer MA, Swedberg K (2003). Effects of candesartan in patients with chronic heart failure and preserved left-ventricular ejection fraction: the CHARM-Preserved Trial. Lancet.

[15] Massie BM, Carson PE, McMurray JJ (2008). Irbesartan in patients with heart failure and preserved ejection fraction. N Engl J Med.

[16] Edelmann F, Wachter R, Schmidt AG (2013). Effect of spironolactone on diastolic function and exercise capacity in patients with heart failure with preserved ejection fraction: the Aldo-DHF randomized controlled trial. JAMA.

[17] Pitt B, Pfeffer MA, Assmann SF (2014). Spironolactone for heart failure with preserved ejection fraction. N Engl J Med.

[18] Liu F, Chen Y, Feng X, Teng Z, Yuan Y, Bin J (2014). Effects of beta-blockers on heart failure with preserved ejection fraction: a meta-analysis. PLoS One.

[19] Zile MR, Baicu CF, Gaasch WH (2004). Diastolic heart failure--abnormalities in active relaxation and passive stiffness of the left ventricle. N Engl J Med.

[20] Lam CS, Roger VL, Rodeheffer RJ, Borlaug BA, Enders FT, Redfield MM (2009). Pulmonary hypertension in heart failure with preserved ejection fraction: a community-based study. J Am Coll Cardiol.

[21] McDonagh TA, Metra M, Adamo M (2021). 2021 ESC guidelines for the diagnosis and treatment of acute and chronic heart failure. Eur Heart J.

[22] Fukuta H, Goto T, Wakami K, Ohte N (2016). Effects of drug and exercise intervention on functional capacity and quality of life in heart failure with preserved ejection fraction: a meta-analysis of randomized controlled trials. Eur J Prev Cardiol.

[23] Zile MR, Brutsaert DL (2002). New concepts in diastolic dysfunction and diastolic heart failure: part II: causal mechanisms and treatment. Circulation.

[24] Aurigemma GP, Gaasch WH (2004). Clinical practice. Diastolic heart failure. N Engl J Med.

[25] Redfield MM (2004). Understanding "diastolic" heart failure. N Engl J Med.

[26] Nishio M, Sakata Y, Mano T (2008). Beneficial effects of bisoprolol on the survival of hypertensive diastolic heart failure model rats. Eur J Heart Fail.

[27] Komajda M, Böhm M, Borer JS, Ford I, Tavazzi L, Pannaux M, Swedberg K (2018). Incremental benefit of drug therapies for chronic heart failure with reduced ejection fraction: a network meta-analysis. Eur J Heart Fail.

[28] Kuno T, Ueyama H, Fujisaki T, Briasouli A, Takagi H, Briasoulis A (2020). Meta-analysis evaluating the effects of renin-angiotensin-aldosterone system blockade on outcomes of heart failure with preserved ejection fraction. Am J Cardiol.

[29] Lin Y, Wu M, Liao B, Pang X, Chen Q, Yuan J, Dong S (2021). Comparison of pharmacological treatment effects on long-time outcomes in heart failure with preserved ejection fraction: a network meta-analysis of randomized controlled trials. Front Pharmacol.

